# Monitoring Dolphins in an Urban Marine System: Total and Effective Population Size Estimates of Indo-Pacific Bottlenose Dolphins in Moreton Bay, Australia

**DOI:** 10.1371/journal.pone.0065239

**Published:** 2013-06-03

**Authors:** Ina C. Ansmann, Janet M. Lanyon, Jennifer M. Seddon, Guido J. Parra

**Affiliations:** 1 Marine Vertebrate Ecology Research Group, School of Biological Sciences, The University of Queensland, St Lucia, Queensland, Australia; 2 School of Veterinary Science, The University of Queensland, Gatton, Queensland, Australia; 3 Cetacean Ecology, Behaviour and Evolution Lab, School of Biological Sciences, Flinders University, Adelaide, South Australia, Australia; 4 South Australian Research and Development Institute (SARDI), Aquatic Sciences, West Beach, South Australia, Australia; University of Bologna, Italy

## Abstract

Moreton Bay, Queensland, Australia is an area of high biodiversity and conservation value and home to two sympatric sub-populations of Indo-Pacific bottlenose dolphins (*Tursiops aduncus*). These dolphins live in close proximity to major urban developments. Successful management requires information regarding their abundance. Here, we estimate total and effective population sizes of bottlenose dolphins in Moreton Bay using photo-identification and genetic data collected during boat-based surveys in 2008–2010. Abundance (*N*) was estimated using open population mark-recapture models based on sighting histories of distinctive individuals. Effective population size (*N_e_*) was estimated using the linkage disequilibrium method based on nuclear genetic data at 20 microsatellite markers in skin samples, and corrected for bias caused by overlapping generations (*N_e_*c). A total of 174 sightings of dolphin groups were recorded and 365 different individuals identified. Over the whole of Moreton Bay, a population size *N* of 554±22.2 (SE) (95% CI: 510–598) was estimated. The southern bay sub-population was small at an estimated *N* = 193±6.4 (SE) (95% CI: 181–207), while the North sub-population was more numerous, with 446±56 (SE) (95% CI: 336–556) individuals. The small estimated effective population size of the southern sub-population (*N_e_*c = 56, 95% CI: 33–128) raises conservation concerns. A power analysis suggested that to reliably detect small (5%) declines in size of this population would require substantial survey effort (>4 years of annual mark-recapture surveys) at the precision levels achieved here. To ensure that ecological as well as genetic diversity within this population of bottlenose dolphins is preserved, we consider that North and South sub-populations should be treated as separate management units. Systematic surveys over smaller areas holding locally-adapted sub-populations are suggested as an alternative method for increasing ability to detect abundance trends.

## Introduction

Successful conservation strategies require detailed biological knowledge of the target species [1]. Accurate assessments of population size and trends in abundance of target species are particularly important for detection of impacts and threats, as well as design and assessment of marine protected areas. For small populations, management also needs an understanding of the effective population size (*N_e_*), which is generally interpreted as an indication of the number of breeding individuals and may be much smaller than the total number of adults present in the population [2]. A small effective population size indicates that the population may be at high risk of losing genetic variation, for example through genetic drift or inbreeding [3].

Moreton Bay (27°S, 153°E; ∼1,400 km^2^) in Queensland, Australia, lies close to greater Brisbane, a major urban centre with one of the highest rates of human population growth in the world [4]. The population size of Brisbane is projected to double from 1.8 million in 2004 to around 3.4 million in 2051, when almost half the population of Queensland will be living in the capital city, and immigration from overseas as well as other Australian states continues to increase [5]. Situated in an area of overlap between tropical and temperate ecosystems, Moreton Bay is home to a large diversity and high biomass of marine wildlife, including many endemic species [6], [7]. It also supports several significant populations of marine megafauna including 14 marine mammal species that occur infrequently or migrate through the area, as well as three resident species of marine mammals: the dugong, *Dugong dugon*, the Indo-Pacific humpback dolphin, *Sousa chinensis*, and the inshore bottlenose dolphin, *Tursiops aduncus* [8].

Large populations of megafauna are becoming increasingly rare in coastal habitats that are subject to increasing human activities [9]. Top predators such as dolphins serve important ecological functions and their declines can have direct as well as indirect effects on a range of other species (including commercially valuable ones) within their ecosystem [10], [11]. Further, marine mammals can serve as indicator species of ecosystem health and as flagship species to raise support for conservation and management efforts [12]. With the declaration of the Moreton Bay Marine Park (MBMP) in 1993, an effective framework for the management and protection of the bay’s resources was established [13], [8]. The MBMP Zoning Plan, which divides the MBMP into general use, habitat, conservation and national park protection zones plus a number of designated protection areas (e.g., for turtles and dugongs) to which special rules apply, was recently reviewed, with the new plan (effective March 2009) increasing the number and size of protection zones from 0.5% to 16% of the MBMP area [14]. However, the marine park currently makes no provisions for the conservation of the two dolphin species that reside in the area. As Moreton Bay is impacted by increasing coastal development including loss of nearshore habitat, declining water quality and increased boating and fishing [6], monitoring of large marine apex predators such as dolphins is required to address future conservation and management needs.

**Figure 1:**
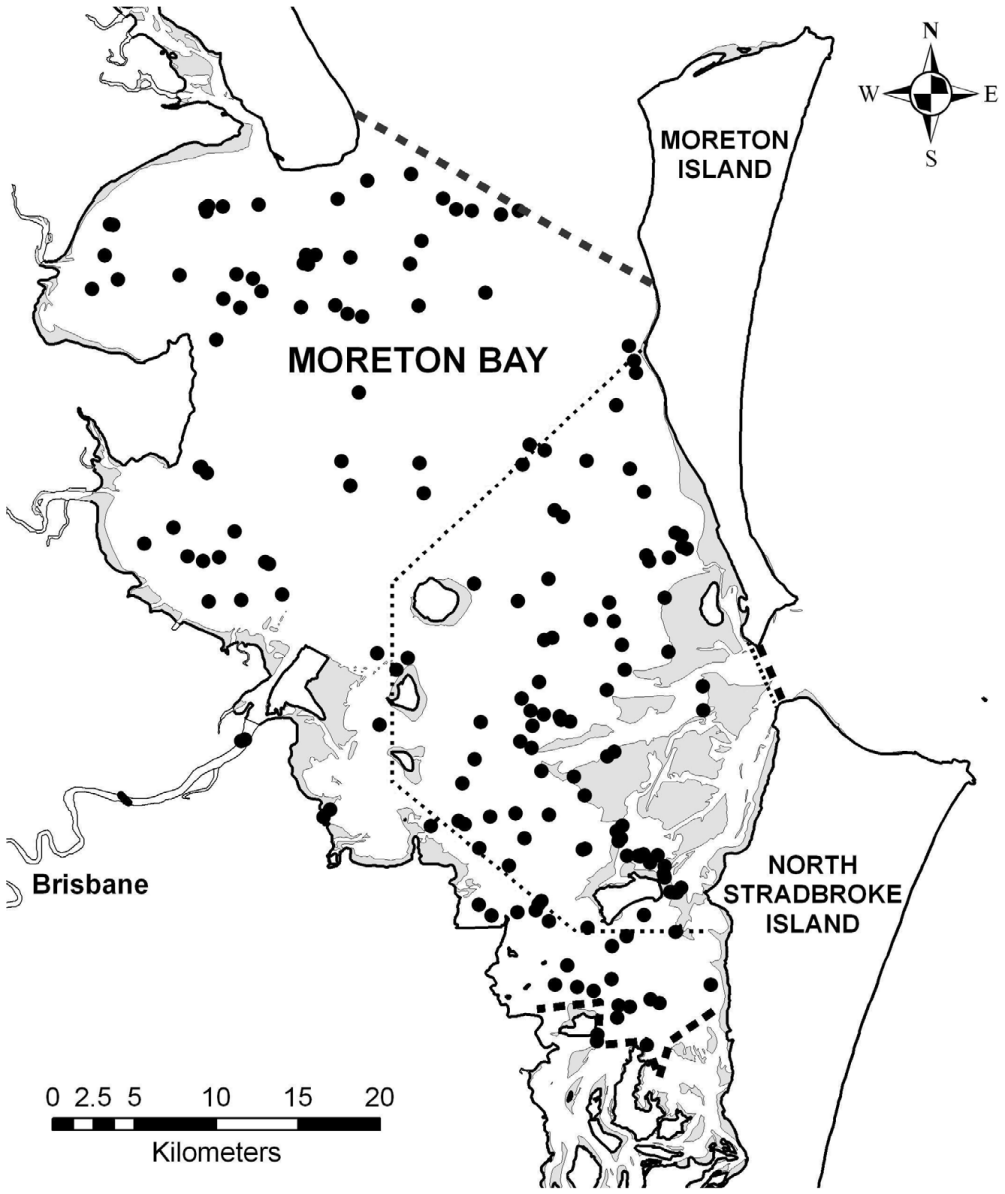
Study areas and sighting locations of bottlenose dolphins in Moreton Bay. Map of Moreton Bay showing sighting locations of bottlenose dolphin schools (black dots) within the current ∼1,300 km^2^ study area (dashed lines) compared to study area covered by previous mark-recapture abundance estimates in 1997/98 (dotted lines) (Chilvers 2001; Lukoschek & Chilvers 2008). Shaded areas indicate shallow water/sandbanks.

Despite their close proximity to a highly urbanised coast, few studies have focused on the bottlenose dolphin population in Moreton Bay [15]–[18] and none over the past decade. Recent findings indicate that bottlenose dolphins in Moreton Bay are divided into two genetically and ecologically divergent sub-populations, one found in shallow nearshore areas of the southern bay and the other in deeper open waters of northern-central Moreton Bay [19], [20]. The only available population size estimates of 600–800 bottlenose dolphins are from surveys conducted over a decade ago that covered only a relatively small study area (∼350 km^2^) in the central-eastern part of the bay [21], which overlapped the distributions of both recently identified sub-populations (Figure 1). No abundance estimates for the entire Moreton Bay area or for each of the two divergent sub-populations are currently available. Therefore available population size estimates are out-dated and no longer appropriate for management purposes. Furthermore, the southern sub-population, in particular, has raised potential concerns as it is found in a restricted and increasingly developed part of the bay, is exposed to higher levels of heavy metal contaminants including lead [19] and shows low genetic diversity and high relatedness [20], suggesting that its effective population size may be low or decreasing.

In this study we use sighting histories of individual animals from 2008–2010 and mark-recapture population models to estimate the abundance of bottlenose dolphins for the entire Moreton Bay, as well as for each of the two sub-populations occurring in this area. We also use nuclear genetic data obtained from remote biopsy samples of live dolphins to estimate effective population sizes based on linkage disequilibrium. Implications for the management of bottlenose dolphins within the Moreton Bay Marine Park are discussed, including recommendations regarding the frequency and coverage of monitoring surveys required to detect population declines.

## Materials and Methods

### Ethics Statement

Data were collected under permits from the Queensland Government Environmental Protection Agency (WITK04729707), the Queensland Parks and Wildlife Service (QS2008/CVL1413) and under approval by the University of Queensland Animal Ethics Committee (SVS/622/08/OPCF and SVS/350/10/WV SCOTT FOUNDATION).

### Data Collection

**Figure 2:**
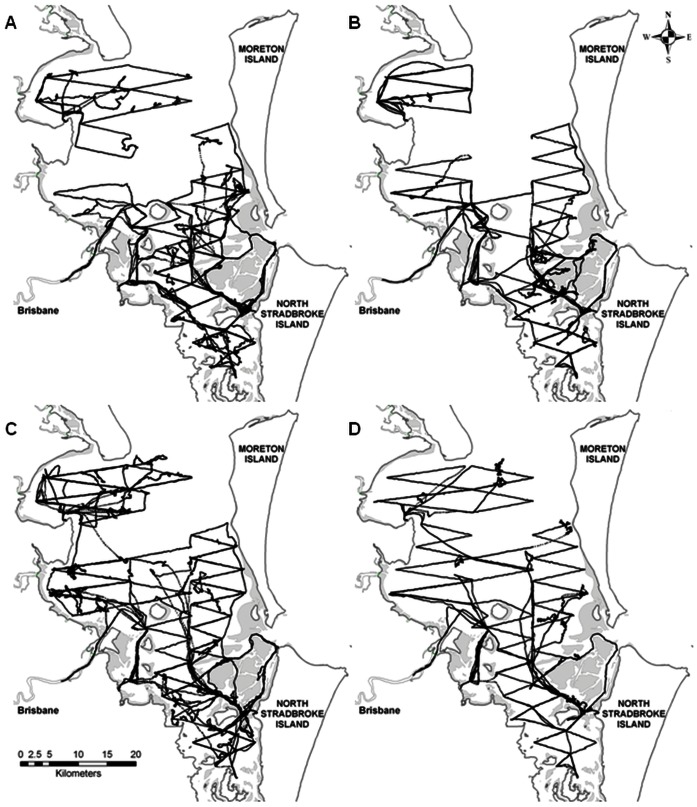
Survey effort across four field seasons in Moreton Bay. GPS track records of boat survey effort in Moreton Bay during four field seasons: A) July–September 2008, B) January–March 2009, C) July–September 2009, D) January–March 2010.

Regular systematic boat-based surveys of bottlenose dolphins were carried out in Moreton Bay (27°00′–27°35′S, 153°00′–153°27′E) over a total of 86 survey days. Surveys were conducted throughout four field seasons, each of three months duration, over two years: 24 days in austral winter (July-September) 2008, 21 in summer (January-March) 2009, 26 in winter 2009 and 15 in summer 2010. Boat surveys followed pre-determined zigzag line transects designed to optimise sampling coverage of all areas and habitat types within Moreton Bay, covering an area of approximately 1,300 km^2^ (Figure 2). Surveys were run at a speed of 10–12 km h^−1^ using a 5.8 m powerboat with a 75 kW outboard engine. Surveys were conducted in Beaufort sea states ≤4, and in daylight between 0630 and 1800 hours. Upon encountering bottlenose dolphins, survey effort was suspended to collect data on location (using GPS), water depth (using depth sounder) and school size and age composition. Dolphins were categorised as adults (>2 m body length [22]), juveniles (∼^2^/_3_ of adult size and not closely associated with a particular adult) or dependent calves (<^2^/_3_ of adult size and closely associated with an adult, the presumed mother). A school was defined as a group of individuals within a ∼100 m radius area and showing similar or coordinated behavior after Wells et al [23] and Lusseau et al [24].

During sightings, we attempted to photographically identify every dolphin within each school, using a Canon EOS 400 D digital SLR camera with a Canon 90–300 mm zoom lens. Only high-quality photographs (based on focus, contrast, angle and distance to animal) were used to identify individuals based on the size, shape, location and pattern of notches on the trailing and leading edges of the dorsal fin, and dorsal and lateral body markings [25], [26]. Skin and blubber biopsy samples were collected from adult dolphins using the PAXARMS biopsy system [27]. DNA was extracted from skin samples for genotyping at 20 microsatellite markers [28]–[31] and individuals were assigned to genetic sub-populations based on Bayesian clustering [32] (see [20] for full genetic methods).

### Population Assignment

**Figure 3:**
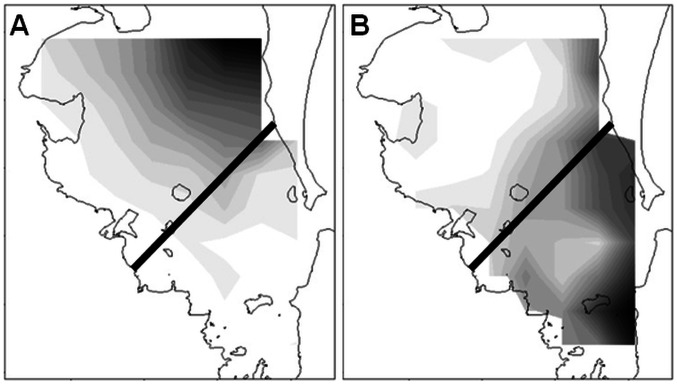
Assignment of individual bottlenose dolphins to sub-populations based on sighting density. Maps of sighting density of bottlenose dolphin sub-populations, A) North and B) South, in Moreton Bay, as predicted by generalised linear modelling (see Ansmann 2011). Individuals that could not be assigned genetically were assigned based on whether their mean sighting location fell north or south of the black diagonal line.

Previous research found that members of one genetic sub-population (North) of bottlenose dolphins were mostly seen in the northern parts of Moreton Bay while the other sub-population (South) was mostly found in the south-east [20]. Further, spatial habitat modelling, stable isotope and trace element analysis confirmed strong resource partitioning (habitat, diet) between these two sub-populations that translated to geographical location [19]. Consequently, sighting location was used to categorise individuals that could not be assigned genetically to North and South populations. This included individuals that were not biopsy sampled, as well as those with ‘mixed’ genetic background and/or weak genetic assignment strength [20]. For this purpose, a southwest – northeast line was delineated on a map of Moreton Bay, based on sighting density distribution of each sub-population as predicted by generalised linear modelling (Figure 3; [19]), and the mean latitude and longitude of all sightings of each photo-identified individual was plotted. If an individual’s mean sighting location fell southeast of the line, it was assigned to the South sub-population, otherwise it was assigned to the North sub-population. For individuals sighted once, that single location was used for assignment. Given that it was not possible to sample and/or assign all individuals genetically, we acknowledge that some individuals may have been placed incorrectly in the wrong sub-population using this method. However, the strong resource partitioning, and fine-scale genetic structure detected between these two sub-populations [19], [20] indicates that such assignment biases are likely to be negligible.

### Total Population Size Estimates

Genetic analyses indicate that low levels of gene flow exist between dolphins within northern Moreton Bay and those found outside the bay area [20], suggesting that some immigration and emigration occurs. Consequently, we used open population Jolly-Seber models for analysis of mark-recapture data [33], [34], which allow for births/deaths or migration. The estimation of demographic parameters under Jolly-Seber capture-recapture models requires a number of assumptions about the nature of the population and the sampling of individuals. Violations of these assumptions can lead to bias in population estimates, making it important to assess and validate each assumption [35]. Assumptions include: 1) marked animals are recognised with certainty and marks are neither lost nor overlooked, 2) sampling periods are instantaneous (i.e., population size does not change during sampling sessions), 3) marked animals have the same probability of being recaptured as unmarked animals (i.e., no behavioural responses to capture), 4) all emigration from the sample area is permanent, 5) every animal in the population has the same probability of capture in a given sampling period and 6) every marked animal has the same probability of survival between sampling periods.

To meet the assumption that marked individuals were reliably recognised, only high quality photographs were used to identify individuals and these were matched by a single experienced investigator (ICA) to avoid observer bias. The main features used for identification included nicks in the dorsal fin which are generally long-lasting [25], pigmentation patterns and/or dorsal fin shape. Regular sampling over a relatively short period of two years permitted comprehensive monitoring of marked animals. Additionally, analysis was restricted to recognisable individuals only, to meet the assumption that all individuals were identifiable, and population size estimates were then adjusted for estimated proportion of non-identifiable individuals, as described below. Each sampling occasion (field season) was relatively short (three months) in comparison to a bottlenose dolphin’s life span of several decades, thus sampling can be considered instantaneous (i.e., population size does not change during sampling sessions). We attempted to reduce heterogeneity in capture probabilities by photographing all individuals within a school, regardless of whether individuals were well marked or not. High capture probabilities (>0.5) can reduce the effects of heterogeneity of capture probabilities on abundance estimates [36]. Negative bias in abundance estimates is expected if average capture probabilities are relatively low (<0.5) [37]. Average capture probabilities among marked animals were relatively high (>0.5), thus we expect the effect of heterogeneity on abundance estimates to be small [35], [37]. Heterogeneous capture or survival probabilities can be caused by temporal emigration (transience) of individuals in the study area [38] and behavioural responses of animals to being “marked” (trap-response) [39]. We used goodness-of-fit tests implemented in the program U-CARE version 2.3.2 [40] to test for any violations of the assumptions of homogeneity of capture and survival probabilities, transience and trap-response. These goodness-of-fit tests have been found to have low power; thus their results should be interpreted with the biology of the target species and sampling design in mind [41].

To estimate the abundance of marked animals (*N*_marked_) for the whole of Moreton Bay as well as for each of the two separate sub-populations, we analysed the mark-recapture data of all photographically identified adult and independent juvenile dolphins using Schwarz and Arnason’s [42] parameterisation of the Jolly-Seber open population model implemented in the POPAN submodule of the program MARK version 6.1 [43]. Encounter histories of each individual were pooled by field season, giving a total of four capture occasions. Four different open population models were run, which varied in whether the survival probability (φ) and/or capture probability (p) parameters were considered constant (.) or variable through time (t). The appropriate model was selected using the Akaike Information Criterion corrected for small sample size (AICc). If models differed in AICc by less than two units, they were considered to fit the data equally well [44].

To estimate the total abundance of bottlenose dolphins in Moreton Bay, we determined the proportion of distinguishable individuals (θ) from a random sample of 400 high quality photographs (100 from each field season) [45], [21]. The total population size (*N*_total_), accounting for unidentifiable individuals, was calculated as:
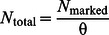



The variance (var) and the standard error (SE) were calculated as:
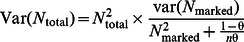


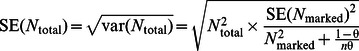
where *n* = the total number of animals from which θ was estimated, i.e., the sample of photographs (*n* = 400) [45], [21]. Confidence intervals for the total population estimates were calculated by assuming the error distribution was the same as for the estimated models with the lower and upper confidence limits the equivalent number of standard errors away from the estimate [after 18].

### Effective Population Size Estimates

Effective population size (*N_e_*) was estimated based on the rate of linkage disequilibrium caused by random processes, using genetic data at 20 microsatellite loci from biopsy skin samples collected from adult bottlenose dolphins (see [20] for genetic methodology). A total of 98 different dolphins were sampled in surveys across Moreton Bay. Of these, 47 individuals were genetically assigned to the North sub-population including 22 samples with high assignment strength (Q>80%). The other 51 samples were assigned to the South sub-population, with 32 of these strongly assigned. Samples were collected over a time period of two years, i.e., representing only one generation. Thus, the linkage disequilibrium method as implemented in the software LDNe version 1.31 [46], which includes a correction for bias caused by low sample sizes less than the true *N_e_* [47], was used to estimate effective population size across all of Moreton Bay (using all 98 individual DNA samples). Two estimates of *N_e_* were generated for each of the two sub-populations, one using only samples of individuals strongly genetically assigned (n_North_ = 22, n_South_ = 32), and another using all genetically assigned individuals regardless of assignment strength (n_North_ = 47, n_South_ = 51). Using only strongly assigned individuals may artificially reduce linkage disequilibrium and thus overestimate *N_e_*, while including admixed (weakly assigned) individuals may increase linkage disequilibrium and cause negative bias to *N_e_* [48]. Thus, we generated these two estimates for each sub-population to give a range of *N_e_* that may approximate true *N_e_*. The critical allele frequency cut-off (P_crit_) was set at 0.05 (lowest allele frequency used) for all estimates except that of strongly assigned North dolphins, as this level is considered to have the least bias for sample sizes >25 (Waples pers. comm. 2013). For the strong North estimate (n = 22), P_crit_ was set at 0.03 to ensure that single copy alleles (at a frequency of 1/44 = 0.023) were screened out [49].

An underlying assumption of the linkage disequilibrium method is that the target species has discrete generations [50]. This assumption is violated in many wildlife species [51] including bottlenose dolphins which are long-lived and have a polygamous mating system with strongly overlapping generations: common bottlenose dolphins, *T. truncatus*, have been recorded to live to over 45 years of age and reach sexual maturity at 5–14 years, while *T. aduncus* appear to reach maturity at a slightly later age of at least 12 years [52]. Female *T. aduncus* tend to breed on average every four years with a gestation period of about one year, followed by a lactation period of 1–5 years [53]. Robinson & Moyer [51] concluded that the best estimates of *N_e_* are obtained by random sampling of mature individuals, which is the case in this study. However, the magnitude of the bias caused by overlapping generations depends strongly on the species’ life history characteristics [51]. It has been found that for samples from mature adult bottlenose dolphins (*Tursiops truncatus*) of different ages, the *N_e_* estimate obtained using LDNe with a P_crit_ of 0.05 is generally biased downwards by about 25% while P_crit_ of 0.02 gave a downward bias of 10–15% (Waples pers. comm. 2013). The magnitude of bias at a P_crit_ of 0.03 has not been examined empirically, thus we assumed this to fall between the values of P_crit_ = 0.05 and 0.02 at around 20%. Given that a number of interacting factors determine actual bias, we acknowledge that this is an approximation and has not been empirically validated. To give a corrected, unbiased estimate of effective population size (*N_e_*c), we adjusted our estimates accordingly by adding 1/3 for estimates derived with P_crit_ = 0.05 and adding 1/4 for the North, strong estimate using P_crit_ = 0.03.

### Power to Detect Population Trends

Gerrodette’s [54] inequality model was used to investigate how many annual surveys would be needed to detect population trends in abundance of bottlenose dolphins in Moreton Bay:
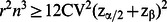
where *r* = the rate of population change, *n* = the number of estimates or monitoring surveys conducted, CV = the coefficient of variation of the estimate of population size, *z_α/2_* =  the one-tailed probability of making a Type I error and *z_β_* = the probability of making a Type II error. The probabilities of Type I and II errors were set at 0.05. The range of CV values obtained from population size estimates was used to assess the effect of different levels of precision on the number of surveys required to detect different rates of population change with high statistical power.

## Results

### Survey Effort and Photo-identification

**Figure 4:**
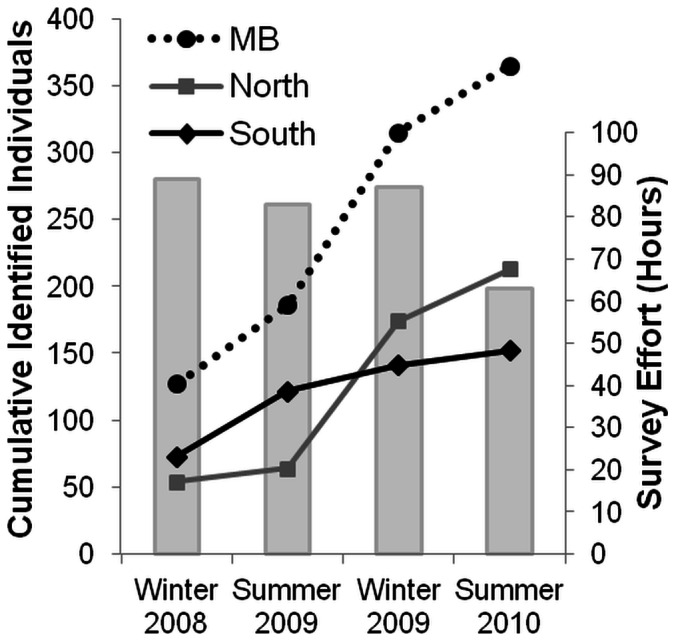
Survey effort and identification curves for bottlenose dolphin sub-populations in Moreton Bay. Cumulative number of identified individual bottlenose dolphins over four field seasons (July-September 2008, January-March 2009, July-September 2009, January-March 2010) in Moreton Bay (MB; dotted black line), and North (grey line) and South (black line) sub-populations, as well as survey effort (hours; grey bars).

Throughout the three-month field seasons, 89 hours of survey effort were expended in winter 2008, 83 hours in summer 2009, 87 hours in winter 2009 and 63 hours in summer 2010 (Figure 4). During the first three seasons, most of the study area was surveyed twice per season. In the last summer season (2010), most of the area was surveyed once only, due to continual poor weather conditions (Beaufort sea state ≥4 and/or rain) (Figure 2). The northern-central areas of Moreton Bay were surveyed less frequently, because these areas are larger, more open and experience large swells and generally worse weather conditions (especially during summer) than the sheltered southern bay. Further, due to logistic constraints relating to the locations of boat launch sites, we could not cover the far north-eastern Moreton Bay area (Figure 2).

**Figure 5:**
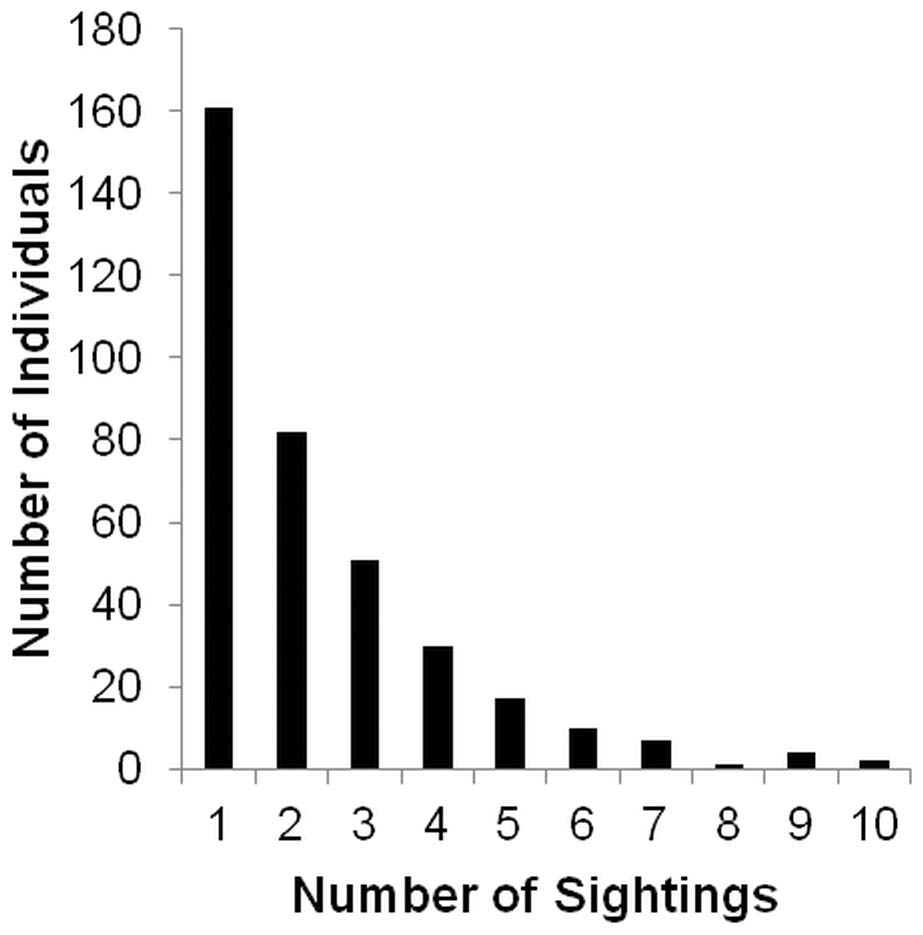
Sighting rate of individual bottlenose dolphins. Total number of individual bottlenose dolphins that were sighted on between one and ten occasions over 2008–2010 in Moreton Bay.

A total of 174 sightings of schools of bottlenose dolphins were recorded in Moreton Bay (Figure 1): 59 in winter 2008, 24 in summer 2009, 72 in winter 2009 and 19 in summer 2010. School size ranged from 1 to 35 dolphins, with a mean ± SE of 6.4±0.51. A total of 365 different individuals were photo-identified throughout Moreton Bay and greater than half of these (204) were sighted more than once, with some individuals recorded on up to ten different occasions (Figure 5). A total of 161 individuals were sighted once only, 82 twice and 51 three times, with a total of 71 dolphins seen four or more times (Figure 5). Biopsy samples were obtained from 98 (27%) of the photo-identified dolphins. All animals biopsied were adult individuals. The analysis of random high quality photographs indicated that the proportion of identifiable individuals (θ) in the sampled population was 0.89.

The identification curve (cumulative number of identified individuals, Figure 4) indicated that out of 365 dolphins identified throughout Moreton Bay, 127 new individuals were added in winter 2008, 59 in summer 2009, 129 in winter 2009 and 50 in summer 2010. For the southern sub-population, the proportion of newly identified individuals decreased with each subsequent field season (Figure 4). After the first two seasons, 122 individuals were identified, with the 49 new individuals photographed during Season 2 making up 40.2% of total marked individuals. During the third and fourth seasons, only 19 (13.5%) and eleven (7.2%) new individuals were added, respectively. This suggests that the total number of 152 individuals that were identified is close to the entire identifiable sub-population in this southern region. In contrast, the proportion of new individuals identified per field season did not decrease consistently for the northern sub-population (15.6% in Season 2, 63.2% in Season 3 and 18.3% in Season 4), suggesting that this sub-population was sampled incompletely. A total of 213 individuals were identified in the northern sub-population (Figure 4).

### Moreton Bay Population Size Estimate

For the identifiable animals over the entire Moreton Bay, goodness-of-fit tests of homogeneity in survival and capture probabilities were significant (Χ^2^ = 18.6, df = 4, *P*<0.001), with no evidence of trap-response (z = −0.6, *P* = 0.54) but significant evidence of transience (z = 3.8, *P*<0.001), suggesting some movement of individuals in and out of the study area (Table 1). This may bias estimates, however, the direction and magnitude of this bias is difficult to predict as it depends on the nature of the emigration process [38] – information that is currently unavailable. The Jolly-Seber model with variable survival and constant capture probability (φ(t)p(.)) had the lowest AICc value of the four models run, yet it was within one AICc point of the fully variable model (φ(t)p(t)). Given the evidence of heterogeneity in capture and survival probabilities, transience and incomplete sampling of the Northern sub-population, we chose the fully variable model as the most appropriate (Table 2). Capture probability was estimated as p = 0.5 (SE = 0.06) and p = 0.58 (SE = 0.05) in seasons 2 and 3 respectively. The population estimate for marked bottlenose dolphins spanning the entire survey period (four seasons) derived from this model was *N*_marked_ = 493±17.8 (SE) (95% CI: 458–528). The total estimate of abundance, adjusting for the proportion of identifiable individuals (θ = 0.89), was *N*_total = _554±22.2 (SE) (95% CI: 510–598) (Table 3).

**Table 1:** Results of goodness-of-fit tests for homogeneity of capture, transience and trap response for sighting history data of identified bottlenose dolphins over the entire Moreton Bay (MB) as well as for each sub-population (South and North) separately.

	Overall Test for Homogenous Capture and Survival Probabilities	Test for Transience	Test for Trap Response
MB	X^2^ = 18.6; df = 4; P<0.001	z = 3.8; P<0.001	z = −0.6; P = 0.54
South	X^2^ = 12.9; df = 4; P<0.05	z = 0.3; P = 0.77	z = −3.1; P<0.01
North	X^2^ = 2.4; df = 3; P = 0.49	z = 1.5; P = 0.14	z = 0; P = 1

**Table 2:** Abundance estimates from the four Jolly-Seber models fitted to mark-recapture data for the entire Moreton Bay (MB) as well as for each sub-population (South and North) over all field seasons.

	Jolly-SeberModel	AICc	Parameters	*N*_marked_ ± SE *(95% CI)*	*N*_total_ ± SE *(95% CI)*	CV(*N*_total_)
MB	φ(t)p(.)	740.83	7	539±25.5 *(489–589)*	606±30.6 *(545–667)*	0.05
	**φ(t)p(t)**	**741.16**	**8**	**493±17.8 ** ***(458–528)***	**554±22.2 ** ***(510–598)***	**0.04**
	φ(.)p(t)	752.34	8	568±35.3 *(499–637)*	638±41.2 *(558–719)*	0.07
	φ(.)p(.)	785.50	5	554±26.1 *(503–605)*	623±31.3 *(561–684)*	0.05
South	**φ(t)p(t)**	**467.81**	**9**	**172±4.8 ** ***(163–182)***	**193±6.4 ** ***(181–207)***	**0.03**
	φ(.)p(.)	468.53	6	179±6.8 *(166–192)*	201±8.4 *(185–217)*	0.04
	φ(t)p(.)	468.88	8	180±7.2 *(165–194)*	202±8.8 *(184–219)*	0.04
	φ(.)p(t)	469.60	8	175±6.0 *(163–186)*	197±7.6 *(182–211)*	0.04
North	φ(.)p(t)	209.50	8	799±205.1 *(397–1,201)*	898±231.0 *(445–1,351)*	0.26
	**φ(t)p(t)**	**210.33**	**9**	**397±49.4 ** ***(300–494)***	**446±56.1 ** ***(336–556)***	**0.13**
	φ(t)p(.)	233.32	6	511±70.2 *(373–648)*	574±79.5 *(418–729)*	0.14
	φ(.)p(.)	270.23	4	627±86.3 *(458–796)*	705±97.8 *(513–896)*	0.14

The fully time-dependent models were selected as most appropriate (highlighted in bold) based on AICc values and evidence of heterogeneity in capture and survival probabilities as well as survey effort.

AICc = corrected Akaike Information Criterion; φ = survival probability; p = capture probability; (t) = time dependent effect; (.) = constant effect; *N*_marked_ = estimated number of marked animals over all field seasons; *N*_total_ = estimated total population size (adjusting for proportion of identifiable individuals).

**Table 3:** Abundance estimates of bottlenose dolphins in Moreton Bay (MB) and sub-populations (South and North) derived from fully time-dependent Jolly-Seber models by field season.

	Field Season	n	p ± SE	φ ± SE	*N*_marked_ ± SE*(95% CI)*	*N*_total_ ± SE*(95% CI)*	CV(*N*_total_)
**MB**	Winter 2008	127	na	na	127±9.5 (108–146)	143±11.0 (121–165)	0.08
(n = 365)	Summer 2009	103	0.50±0.06	0.68±0.06	207±23.7 (161–254)	233±26.9 (180–286)	0.12
	Winter 2009	213	0.58±0.05	1.00±0.00	370±25.6 (320–420)	416±29.7 (358–474)	0.07
	Summer 2010	109	na	0.22±0.03	109±9.1 (91–127)	123±10.5 (102–143)	0.09
	Overall				493±17.8 (458–528)	554±22.2 (510–598)	0.04
**South**	Winter 2008	73	na	na	73±6.3(61–85)	82±7.2(68–96)	0.09
(n = 152)	Summer 2009	90	0.75±0.07	0.75±0.06	120±10.1(100–140)	135±11.6(112–158)	0.09
	Winter 2009	78	0.60±0.07	0.96±0.09	130±13.6(103–156)	146±15.5(115–176)	0.11
	Summer 2010	61	na	0.43±0.06	61±6.1(49–73)	69±7.0(55–82)	0.10
	Overall				172±4.8 (163–182)	193±6.4 (181–207)	0.03
**North**	Winter 2008	54	na	na	54±6.7 (41–67)	61±7.6 (46–75)	0.13
(n = 213)	Summer 2009	12	0.06±0.04	0.61±0.17	195±128.9 (0–448)	219±144.9 (0–504)	0.66
	Winter 2009	135	0.62±0.26	0.95±0.42	219±91.2 (40–397)	246±102.6 (45–446)	0.42
	Summer 2010	48	na	0.06±0.02	48±6.3 (36–60)	54±7.1 (40–68)	0.13
	Overall				397±49.4 (300–494)	446±56.1 (336–556)	0.13

n = number of identified individuals; p = capture probability; φ = survival probability; *N*_marked_ = estimated abundance of marked animals; *N*_total_ = estimated total population size (adjusting for proportion of identifiable individuals); na = not available.

### Sub-population Size Estimates

Goodness-of-fit tests found significant heterogeneity in survival and capture probabilities (Χ^2^ = 12.9, df = 4, *P*<0.05), with no evidence of transience in the southern sub-population (z = 0.3, *P* = 0.77) but significant trap-shyness (z = −3.1, *P*<0.01) (Table 1), which may cause an upwards bias in population size estimates [35]. For the POPAN mark-recapture analysis of the southern sub-population, AICc values of all four models differed by less than two units and should thus be considered equally good models to explain the data. Based on AICc, the fully variable model (φ(t)p(t)) was selected as the best fitting model (Table 2). Capture probabilities were relatively high at p = 0.75 (SE = 0.07) and p = 0.6 (SE = 0.07) in seasons 2 and 3 respectively. The marked population estimate (across all seasons) derived from this model for the southern sub-population was *N*_marked_ = 172±4.8 (SE) (95% CI: 163–182). Adjusting this estimate by the proportion of identifiable individuals (θ = 0.89) gave a total population estimate of *N*_total_ = 193±6.4 (SE) (95% CI: 181–207) (Table 3). Abundance estimates from the other three (competing) models were similar (Table 2).

For the northern sub-population, goodness-of-fit tests found no evidence of heterogeneity in survival and capture probabilities (Χ^2^ = 2.4, df = 3, *P* = 0.49), transience (z = 1.5, *P* = 0.14) nor trap-response (z = 0, *P* = 1) (Table 1). The model with constant survival and variable capture probability (φ(.)p(t)) had the lowest AICc, however, AICc for the fully time-dependent model (φ(t)p(t)) was only marginally higher (difference <1), indicating that this model fit the data equally well [44]. Given the incomplete sampling of the Northern sub-population suggested by the identification curve (see Figure 4), lower survey effort in the North and known low power of goodness of fit tests, we chose the fully variable model (φ(t)p(t)) as the most appropriate (Table 2). Capture probability for the northern sub-population was extremely low in summer 2009, p = 0.06 (SE = 0.04), but high in winter 2009, p = 0.62 (SE = 0.26), reflecting the heterogeneous survey coverage in this part of the bay, which was most likely related to weather conditions (generally better in winter than in summer). The population estimate of marked individuals derived from the fully time-dependent model was *N*_marked_ = 397±49.4 (SE) (95% CI: 300–494) equating to an estimated total population size *N*_total_ = 446±56.1 (SE) (95% CI: 336–556) for the northern sub-population (Table 3).

### Effective Population Size Estimates

Using the genetic linkage disequilibrium method, total effective population size corrected for bias introduced by overlapping populations for bottlenose dolphins over the entire Moreton Bay was estimated as *N_e_*c = 127 (95% CI: 93–185). For the South genetic cluster, estimated *N_e_*c was 56 (95% CI: 33–128) when only the 32 strongly assigned (Q >80%) dolphins were included. When all 51 dolphins assigned to the South cluster were included, estimated *N_e_*c was 75 (95% CI: 48–136). For the North cluster, *N_e_*c was estimated at 473 (95% CI: 91– infinity) based on only strongly assigned individuals, and 168 (95% CI: 95–520) when all North dolphins were included (Table 4). Note that “infinity” in the confidence interval for the first North sub-population estimate should be interpreted as the possibility (within 95% confidence) that there is no evidence for disequilibrium caused by genetic drift due to a finite number of parents, or that *N_e_* is large enough (> ∼500) for such genetic effects to be undetectable [46], [49].

**Table 4:** Estimates of effective population sizes (*N_e_*) based on linkage disequilibrium, calculated using biopsy samples collected across the whole of Moreton Bay (MB), only samples strongly assigned to either of two sub-populations (South and North) or all samples assigned to each sub-population regardless of assignment strength.

	Assignment	n	P_crit_	Independent Comparisons	Overall r^2^	*N_e_*	95% CI	*N_e_*c	corrected 95% CI
MB	na	98	0.05	1750	0.014	95	70–139	127	93–185
South	strong	32	0.05	945	0.042	42	25–96	56	33–128
	all	51	0.05	1127	0.027	56	36–102	75	48–136
North	strong	22	0.03	2502	0.053	378	73–infinity	473	91–infinity
	all	47	0.05	2051	0.025	126	71–390	168	95–520

n = sample size; P_crit_ = lowest allele frequency used; na = not available.

### Detecting Population Trends

**Figure 6:**
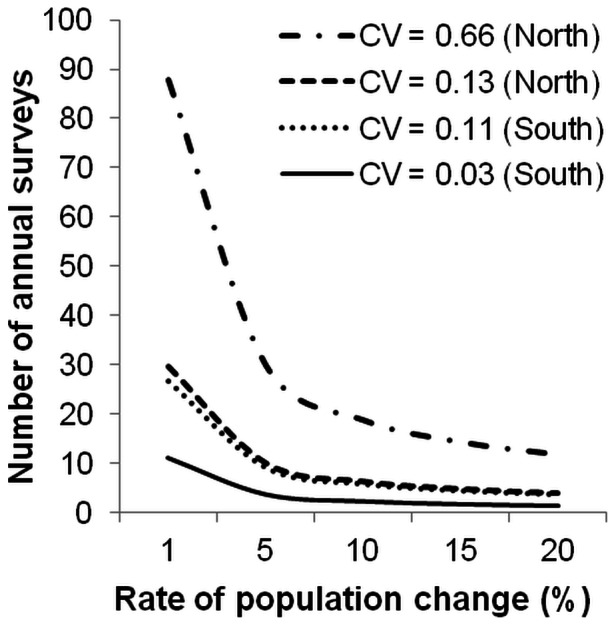
Required surveys to detect abundance trends. Number of annual surveys required to detect different rates of abundance trends at highest and lowest levels of precision (CV) obtained for mark-recapture abundance estimates of bottlenose dolphin sub-populations (North and South) in Moreton Bay. The probabilities of Type I and Type II errors were set at 0.05.

Based on Gerrodette’s [54] model, the number of annual surveys (referring to total survey effort at the scale performed here) required to detect trends in abundance decreases with increasing precision (i.e., decreasing CV) of surveys as well as with increasing rate of population change (Figure 6). Approximately four annual surveys would be required to detect a population trend of 5%, whilst two annual surveys would be sufficient to detect a 20% change at the highest precision recorded for surveys of the South sub-population (CV = 0.03). The much lower precision associated with the abundance estimates of the North sub-population greatly increases the frequency of surveys required to detect population changes, especially at slow rates of change. At the highest precision obtained for the North sub-population estimate (CV = 0.13), it would take approximately ten annual surveys to detect a population trend of 5%, and four annual surveys to detect a 20% change (Figure 6).

## Discussion

### Population Size of Bottlenose Dolphins in Moreton Bay

Knowledge of population size plays a critical role in wildlife management and conservation status assessments. This study provides the first empirical estimates of abundance for bottlenose dolphins across the whole of Moreton Bay as well as for each of the two genetically divergent sub-populations (North and South) inhabiting the bay. It also provides the first estimates of effective population sizes based on nuclear genetic data. Our results indicate that bottlenose dolphins are abundant in Moreton Bay with a total population size estimate of 554±22 (SE) (95% CI: 510–598) individuals for the period 2008–10. The South sub-population is relatively small with an estimated 193±6 (SE) (95% CI: 181–207) individuals, while the North sub-population is estimated to be more numerous, with 446±56 (SE) (95% CI: 336–556) individuals.

Obtaining accurate and precise estimates of the abundance and population trends of cetaceans is inherently problematic as they are cryptic (spend long periods of time underwater) and highly mobile, leading to high uncertainty about abundance estimates and low power for detecting trends or negative impacts [55], [56]. Through a detailed examination of the assumptions involved in mark recapture analyses, we were able to identify potential biases in the estimates of abundance of bottlenose dolphins for Moreton Bay. One of the main assumptions of mark-recapture analysis is homogeneous capture and survival probabilities for all individuals. Violation of this assumption leads to an underestimate in abundance estimates [37]. In general, the average capture probabilities obtained in this study were relatively high (p>0.5) for the total Moreton Bay population, thus we expect the effect of heterogeneity to be relatively low. However, homogeneity of capture probabilities is unlikely to be met in any sampling scheme of free-ranging animals [35], [37] and is violated if sampling coverage is heterogeneous across the study area. Survey effort was not evenly distributed across the whole bay in this study, due to logistic constraints and adverse weather conditions affecting the northern and central areas of Moreton Bay in particular (Figure 2). Therefore, the estimate of abundance of bottlenose dolphins presented here for the whole of Moreton Bay (554±22) should be considered as a minimum population estimate.

The lower and heterogeneous survey coverage in the northern area of the bay is likely to have also affected the size estimate for the North sub-population. Further, we were not able to cover the entire northern Moreton Bay area (i.e., area along the north-western coast of Moreton Island). Thus, it is likely that we did not cover the whole range of the northern sub-population and that individuals may have been moving in or out of the study area, potentially biasing these estimates. The cumulative recapture curve for the northern sub-population did not level out over the study duration, and capture probability was highly variable and relatively low in the North, especially in summer, resulting in abundance estimates with high coefficients of variation. Thus, abundance estimates of the northern sub-population should be interpreted with caution, given the high variability among estimates from competing models.

The population estimate of 193 for the South sub-population may be an overestimate as indicated by evidence for trap-shy behaviour of dolphins in this sub-population. However, capture probability was high at 0.6–0.75 and the proportion of newly identified individuals decreased steadily after the first two field seasons indicating that at least a large proportion of this South sub-population was captured, lending a high level of confidence to the accuracy of this estimate (as supported by low CVs).

### Effective Population Size *N_e_*

Estimates of effective population size (*N_e_*) based on the linkage disequilibrium method have been shown to be reliable with use of 10–20 microsatellite loci and samples of at least 25–50 individuals, if the effective population size is small (i.e., less than approximately 500 individuals) [49]. Estimating *N_e_* of larger populations is difficult because of the weak genetic signal relative to sampling noise [49]. In this study, a sufficiently large number of microsatellite loci (20) was used to estimate *N_e_* from sample sizes above or close to the number recommended by Waples & Do [49]. However, it should be noted that the confidence intervals of these estimates are based on the assumptions of the model being met. As discussed above, this is unlikely to be the case here due to population structuring and overlapping generations. Thus, the confidence intervals reported here might not capture the full range of uncertainty (Waples pers. comm. 2013).

Previous findings [19] suggest that the majority of dolphins with weak assignment strength (i.e., mixed genetic background) used habitat and resources similar to the North sub-population, suggesting that the majority of these individuals may integrate functionally with the northern dolphins. Thus, the majority of gene flow between the two sub-populations is probably directional from South to North, as also suggested by genetic patterns of sex-biased dispersal and diversity [20]. Therefore, true *N_e_* for the South sub-population may be closer to the lower estimate generated using only strongly assigned individuals (*N_e_*c = 56; 95% CI: 33–128), whereas it may be more appropriate to include weakly assigned individuals in the estimation of *N_e_* for the North sub-population giving *N_e_*c = 168 (95% CI: 95–520).

The estimated *N_e_*c of 127 (95% CI: 93–185) for the entire Moreton Bay population appears low, especially compared to the individual estimates for both sub-populations. Linkage disequilibrium models assume a closed and unstructured population [47]. Sampling from two sub-populations violates this assumption and may increase disequilibrium above that caused by factors related to effective population size, such as drift. Thus, the *N_e_* estimate for the entire Moreton Bay population may be underestimated by admixture between sub-populations [48].

### Monitoring Abundance Trends

Previous estimates of abundance for bottlenose dolphins in Moreton Bay are over a decade old (from 1997–98) and encompass only a ∼350 km^2^ area in the central-eastern bay [21], an area overlapping the distributions of both North and South sub-populations and only covering approximately 30% of the total ∼1,300 km^2^ area surveyed in the present study. A total abundance of 673±130 SE (95% CI: 606–996) bottlenose dolphins was estimated in 1997 and 818±152 SE (95% CI: 589–1145) in 1998. As previous studies did not cover the whole distributional range of either sub-population, it is impossible to compare previous abundance estimates to those generated here, nor to new estimates for the same ∼350 km^2^ area in the eastern bay, as any changes observed could be caused by shifts in distribution of dolphins over the intervening decade. Thus, in the absence of comparable abundance estimates, population trends of bottlenose dolphins in Moreton Bay cannot be assessed.

Applying the inequality model of Gerrodette [54] indicated that detection of small rates of population change (<5% per year) with high statistical power requires several years of sampling effort. The precision of population estimates has a large effect on the number of annual surveys needed to detect change, especially at low levels [54]. Even at the highest levels of precision achieved here, by the time such rates of decline are detected, population sizes would have decreased significantly. For example, the South sub-population, currently estimated at 193 individuals would be reduced to 157 individuals by the time a 5% decline is detected, in other words, almost a fifth of the population would be lost. Thus, in order to detect trends before populations have declined to critically low numbers, precision should be maximised.

The detection of even precipitous declines (e.g., 50% decrease in abundance) in most whale and dolphin populations is unachievable with present levels of investment into surveys, and current survey technology and design [57]. Improvement of performance in detecting population trends depends on increasing survey effort, development of new methods to detect trends, and/or changes to decision criteria regarding the magnitude and level of evidence needed to establish that a decline is occurring [57]. As increasing survey effort is often logistically difficult and expensive, Taylor *et al.* [57] suggested “trend-site” surveys as an alternative method for increasing ability to detect abundance trends. In this survey design, precision is maximised by surveying more comprehensively over a smaller area. This method makes the assumption that the proportion of the total population surveyed remains constant, i.e., no significant changes of distribution occur among the individuals within this smaller area, or in other words, no animals are moving in and out of the area. This assumption can be met by surveying an area that a demographically distinct sub-population occupies [57]. In the case of bottlenose dolphins in Moreton Bay, the South sub-population is distributed across a restricted area of the southern bay [19], making this area an ideal trend-site for regular monitoring. Further, as this southern sub-population is potentially more vulnerable to decline due to its smaller estimated size, lower genetic diversity [20] and higher exposure to anthropogenic impacts than the North sub-population [19], focussing monitoring effort on the South sub-population would appear to be appropriate. Monitoring programs should also trial the use of alternative methods of estimating abundance, for example aerial line-transect surveys, to investigate whether higher levels of precision could be achieved more efficiently with these methods than the boat-based mark-recapture surveys used in this study.

Another option to monitor population trends is genetic monitoring of effective population size, *N_e_*, which has been suggested as a more reliable means of detecting population declines than estimating abundance, *N*, especially for small populations of *N*<500 [58]. Thus, future survey effort should include genetic sampling and aim to achieve a sample size of at least 60 individuals [58]. However, Tallmon *et al.* [58] also recommend that samples should be collected more than one, preferably at least five generations apart to reliably detect population trends. For a long-lived species like bottlenose dolphins, with relatively long generation times of at least ten years, this again would suggest that a long period may pass before declines are detected. In light of the difficulties associated with reliably monitoring population trends, it is advisable to obtain as much information as possible by combining multiple methodologies and measures, such as estimating both *N* as well as *N_e_* [58].

### Management Implications

Bottlenose dolphins are found both within Moreton Bay and outside the bay off the oceanic side of Moreton and North Stradbroke islands [15]. Our results indicate that the total population of bottlenose dolphins within Moreton Bay (estimated *N* >550 in an area of 1,300 km^2^) is large, particularly for a resident coastal population in close proximity to a major urban centre. For comparison, estimates for much larger embayments (13,000 km^2^) with a relatively undeveloped coastline such as Shark Bay, on the west coast of Australia number in the low thousands, i.e., 2,000–3,000 [59]. Furthermore, outside Moreton Bay off the north-eastern waters of North Stradbroke Island (Figure 1), a population size of between 700 and 1,000 bottlenose dolphins has been identified [60]. Thus, it is clear that Moreton Bay, despite its proximity to one of the fastest growing urban centres in the world, represents an important habitat for bottlenose dolphins.

Although large numbers of bottlenose dolphins inhabit Moreton Bay, this community is composed by two genetically and ecologically distinct subpopulations [19], [20] and such sub-division needs to be considered in future management initiatives. The conservation of locally adapted sub-populations is necessary to ensure that ecological as well as genetic diversity within this population of bottlenose dolphins is preserved. It is well established that smaller populations are more vulnerable to loss of genetic variability through genetic drift, inbreeding or stochastic catastrophic events [61]. While it is difficult to assess the likely persistence of a population, Mace & Lande [62] suggest that a population should be considered at a critical state if its effective population size (*N_e_*) is less than 50. Our estimates for the southern sub-population (*N_e_*c = 56–75) fall just over this critical value. It has to be noted that some genetic exchange does occur between the two sub-populations [20], at a level that may be sufficient for demographic rescue to occur. However, given the lower density of animals and smaller effective population size of the South sub-population, strong resource use differentiation between North and South sub-populations [19], and evidence that intraspecific foraging specializations may lead to limited gene flow between cetacean populations living in sympatry [63], both North and South sub-populations should be considered as relevant entities for management purposes.

The South sub-population of bottlenose dolphins, due to its geographic location, is potentially vulnerable to the additive effects of anthropogenic activities in southern Moreton Bay. This area is subject to high levels of commercial and recreational boating because of its proximity to the Port of Brisbane (Australia’s third busiest port) and the Brisbane River. Further, while commercial fisheries are restricted in this part of the bay by the Moreton Bay Marine Park (MBMP) zoning plan [14], high levels of recreational fishing do occur [64]. This poses threats including entanglement in or ingestion of fishing gear [65] and unregulated feeding of dolphins through discarded bait/fish, which have potential impacts on the health and behaviour of dolphins [66], [67]. Finally, the dolphins in southern Moreton Bay are exposed to higher levels of contaminants including lead, probably through runoff from local catchments and the low levels of mixing with oceanic waters in this almost enclosed part of the bay [19].

In order to effectively conserve the bottlenose dolphins of Moreton Bay, regular monitoring of populations is recommended. Long-term abundance and genetic data from several surveys are required to reliably assess trends. Although ideally these surveys should cover the whole bay to assess numbers and trends for both sub-populations, precision and reliability of trend estimates may be maximised by limiting the survey area to cover the distribution of the more vulnerable South sub-population. In light of ongoing and anticipated urban development in the region, and the high conservation value of Moreton Bay for local bottlenose dolphins, we recommend that strategies be developed to reduce the impacts of detrimental human activities, particularly in the southern part of Moreton Bay. Upgrading the protection status of the already existing habitat protection zones (that largely coincide with the distribution of the Southern sub-population) to conservation or national park zones, and/or creating designated protection areas for dolphins that regulate boating and fishing activities, similar to those for dugongs and turtles currently in place within the Moreton Bay Marine Park [14], will be beneficial for the conservation of the ecology and genetic diversity of bottlenose dolphins in this rapidly growing region.
